# Early Postnatal Exposure to a Low Dose of Nanoparticulate Silver Induces Alterations in Glutamate Transporters in Brain of Immature Rats

**DOI:** 10.3390/ijms21238977

**Published:** 2020-11-26

**Authors:** Beata Dąbrowska-Bouta, Grzegorz Sulkowski, Mikołaj Sałek, Magdalena Gewartowska, Marta Sidoryk-Węgrzynowicz, Lidia Strużyńska

**Affiliations:** 1 Laboratory of Pathoneurochemistry, Department of Neurochemistry, Mossakowski Medical Research Centre, Polish Academy of Sciences, 5 Pawińskiego Street, 02-106 Warsaw, Poland; bbouta@imdik.pan.pl (B.D.-B.); gsulkowski@imdik.pan.pl (G.S.); msalek@imdik.pan.pl (M.S.); msidoryk@imdik.pan.pl (M.S.-W.); 2 Electron Microscopy Research Unit, Mossakowski Medical Research Centre, Polish Academy of Sciences, 5 Pawińskiego Street, 02-106 Warsaw, Poland; mgewartowska@imdik.pan.pl

**Keywords:** nanosilver, neurotoxicity, glutamate transporters, GLAST, EAAC1, GLT-1

## Abstract

Due to strong antimicrobial properties, silver nanoparticles (AgNPs) are used in a wide range of medical and consumer products, including those dedicated for infants and children. While AgNPs are known to exert neurotoxic effects, current knowledge concerning their impact on the developing brain is scarce. During investigations of mechanisms of neurotoxicity in immature rats, we studied the influence of AgNPs on glutamate transporter systems which are involved in regulation of extracellular concentration of glutamate, an excitotoxic amino acid, and compared it with positive control—Ag citrate. We identified significant deposition of AgNPs in brain tissue of exposed rats over the post-exposure time. Ultrastructural alterations in endoplasmic reticulum (ER) and Golgi complexes were observed in neurons of AgNP-exposed rats, which are characteristics of ER stress. These changes presumably underlie substantial long-lasting downregulation of neuronal glutamate transporter EAAC1, which was noted in AgNP-exposed rats. Conversely, the expression of astroglial glutamate transporters GLT-1 and GLAST was not affected by exposure to AgNPs, but the activity of the transporters was diminished. These results indicate that even low doses of AgNPs administered during an early stage of life create a substantial risk for health of immature organisms. Hence, the safety of AgNP-containing products for infants and children should be carefully considered.

## 1. Introduction

Silver nanoparticles (AgNPs) have strong antimicrobial potential and are now included in a wide range of medical and consumer products, some of which are used by infants and children such as food handling devices, baby bottles, pacifiers, toys, blankets, clothes, or cleaning products [1,2]. It has been indicated that such products may cause users to become exposed to AgNPs, potentially causing adverse health effects due to oral or contact exposures [3].

Reports on toxicity of AgNPs in immature organisms have been rare. It should be stressed that the physiology and specific “hand-to-mouth” behavior of infants and children significantly enhance their risk of oral exposure [4,5]. Toxicological studies indicate that, during the pre- and postnatal developmental periods, organisms are generally much more vulnerable to toxicants than in adulthood. It has been shown that neurotoxic effects of AgNPs are inversely related to the age of exposed animals and are strongly exhibited in young organisms [6]. The enhanced susceptibility to harmful substances is the consequence of metabolic immaturity and inefficient detoxifying mechanisms [5].

Among different organs, the brain is particularly vulnerable to stressors. Previous studies on the mechanisms of neurotoxicity of AgNPs have revealed inhibition of axonal outgrowth and degeneration of neurites in cultured cortical neurons [7]. Subcutaneous injections of AgNPs were found to generate pyknotic and necrotic neurons in rat brain [8]. Moreover, prolonged exposure to a low environmentally-relevant dose of AgNPs was found to result in degeneration of synapses, as assessed by ultrastructural and biochemical studies [9]. Although there is no doubt that AgNPs exert neurotoxic effects, the mechanisms of AgNP-induced neuronal injury are not well characterized. It is suspected that neurons are more vulnerable to AgNPs than glial cells, since cultured astrocytes exposed to AgNPs were found to upregulate metallothionein proteins which are protective against metal toxicity [10]. On the other hand, glial cell activation in response to the presence of AgNPs was also identified [11]. Swelling of perivascular astrocytes was also observed in AgNP-exposed animals [12]. In vivo evidence for a toxic influence of AgNPs on glial cells has been scarce. Likewise, it is not known whether the deleterious changes observed in neurons of exposed animals are directly caused by AgNPs or are an indirect result of disturbed neuroprotective function of astroglial cells.

One of the principal functions of astroglia is to maintain proper concentrations of extracellular glutamate, the main excitatory neurotransmitter in the central nervous system (CNS), which is involved in most aspects of brain functions such as cognition, memory, and learning in both adult and developing organisms [13]. Time-dependent concentrations of glutamate in extracellular fluid have an influence on the profile of activation of glutamate receptors and thus determine the post-synaptic response. Accumulation of glutamate in the extracellular space may be toxic to neurons due to over-activation of glutamate N-methyl-D-aspartate receptors (NMDARs) and initiation of downstream signaling pathways leading to excitotoxic cell death (for a review, see [14]). Therefore, extracellular glutamate must be strictly controlled by a group of high affinity glutamate transporters (GluTs) known as: GLAST/EAAT1, GLT-1/EAAT2, EAAC1/EAAT3, EAAT4, and EAAT5 (in rodent/human, respectively). Glutamate released to the synaptic cleft is mainly taken up by transporter systems located in the glial part of the tripartite synapse, where glial glutamate transporter 1 (GLT-1) and glutamate/aspartate transporter (GLAST) are of the great importance.

Considering the fact that the developing brain is highly susceptible to different toxins [15] and that research on the influence of AgNPs on immature brain has been scarce, there is an urgent need to investigate the mechanisms of neurotoxicity induced by AgNPs.

Thus, in the current study, we applied a model of developmental exposure to a low dose of AgNPs. Since previously published data provide indications that AgNPs induce neuronal injury, the current studies were undertaken to search for a contribution of glutamate transporters to this mechanism. We focused on examining the expression and function of three main glutamate transporters: neuronal excitatory amino acid carrier 1 (EAAC1) and two glial transporters, GLAST and GLT-1. Analysis at the level of mRNA and protein expression was combined with immunofluorescence and ultrastructural analyses of brain tissue. The early and late effects of prolonged exposure to AgNPs were assessed at Postnatal Days (PND) 35 and 90, respectively, and compared with effects produced by treatment with ionic silver.

## 2. Results

### 2.1. Temporal Profile of Silver Concentrations in Serum and Brains of Exposed Rats

Both our previous [9] and current studies revealed that small (mean diameter of 10 ± 4 nm) AgNPs obtained from the manufacturer do not aggregate in a citric acid coating buffer. The TEM analysis performed prior to administration of AgNPs revealed that the particles are round and homogenous in size with almost 94% having diameters within 9.0–11.0 nm.

The dose of AgNPs used in the study is relatively close to a theoretically estimated environmental concentration and was chosen based on a published calculation indicating that the predictable no-effect concentration (PNEC) is in the range of 0.04–0.1 mg/L for water compartments [16].

Measurements of silver concentrations in the blood shortly after exposure (PND 35) indicated that the applied dose results in similar ranges of absorption of both silver forms—particulate or ionic (*p* < 0.0001 AgNPs/Ag citrate vs. control) (Table 1). Significant deposition of silver in the brain was observed at PND 35 relative to control, in which it was below the detection limit (*p* < 0.001 vs. control). Moreover, silver concentrations were found to have increased in the brain over the post-exposure time, decreasing concomitantly in the serum of both silver-treated groups relative to the control level. Time-dependent deposition of silver in brain tissue was found to be significantly higher in the Ag citrate group than in the AgNPs group (*p* < 0.001).

### 2.2. Ultrastructural Alterations in Neurons but Not in Astrocytes under Exposure to AgNPs

**Figure 1:**
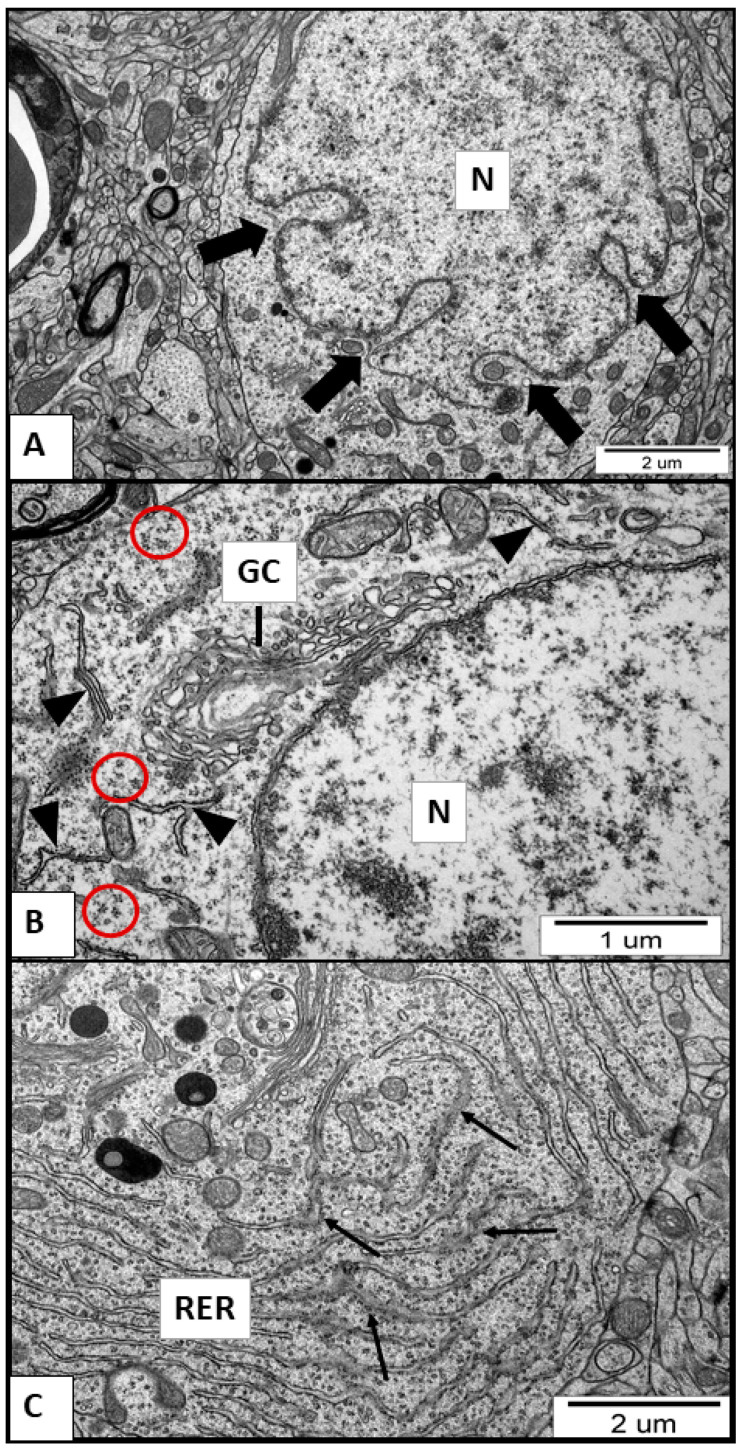
TEM micrographs showing ultrastructural changes in subcellular structures of neurons in brain sections obtained from AgNP-treated rats. (**A**) nuclear changes (N, nucleus) and invaginations of nuclear envelope (thick arrows); (**B**) enlarged and swollen cisternae of Golgi complex (GC), fragments of disintegrated rough endoplasmic reticulum (RER) (arrowheads), and aggregates of ribosomes (red circles); and (**C**) thickened cisternae of RER (thin arrows) and massive detachment of ribosomes lying free in the cytoplasm. Images are representative for each of three animals.

Electron microscopy analysis revealed that neuropil appears normal in specimens obtained from brains of control saline-treated rats. All types of neuronal cells, including neurons and astrocytes, as well as subcellular structures, were properly organized. In TEM images taken from brains of both AgNP- and Ag^+^-treated animals, pathological changes within neurons were found to be present early after the end of exposure (35 PND). Primarily, the nuclear surface was found to be highly irregular. Nuclear envelope invaginations are frequent pathological feature observed in neurons (Figure 1A). Golgi complexes were found to be enlarged and swollen, having intensified vesiculation with formation of abnormal vesicles (Figure 1B). Membranes of the rough endoplasmic reticulum (RER) were thickened, and massive detachment of ribosomes was observed. The detached ribosomes create a granular background in the cytoplasm of affected cells (Figure 1B,C). Degranulation of RER was accompanied by formation of ribosomal aggregates in the neuronal cytoplasm (Figure 1B). In some neurons, the RER structure undergoes disintegration and fragments of thickened membranes were found to be present in cytoplasm (Figure 1B). We did not find similar pathological changes in astrocytes. Furthermore, it appears that the profile of abnormalities observed in neurons does not differ significantly according to the form of silver administered. However, changes were time-dependent and, although still present, were less pronounced at a later point after exposure, i.e., at 90 PND.

### 2.3. Exposure to AgNPs/Ag^+^ Affects the Expression and Functional Activity of Neuronal Excitatory Amino Acid Carrier 1 (EAAC1)

**Figure 2:**
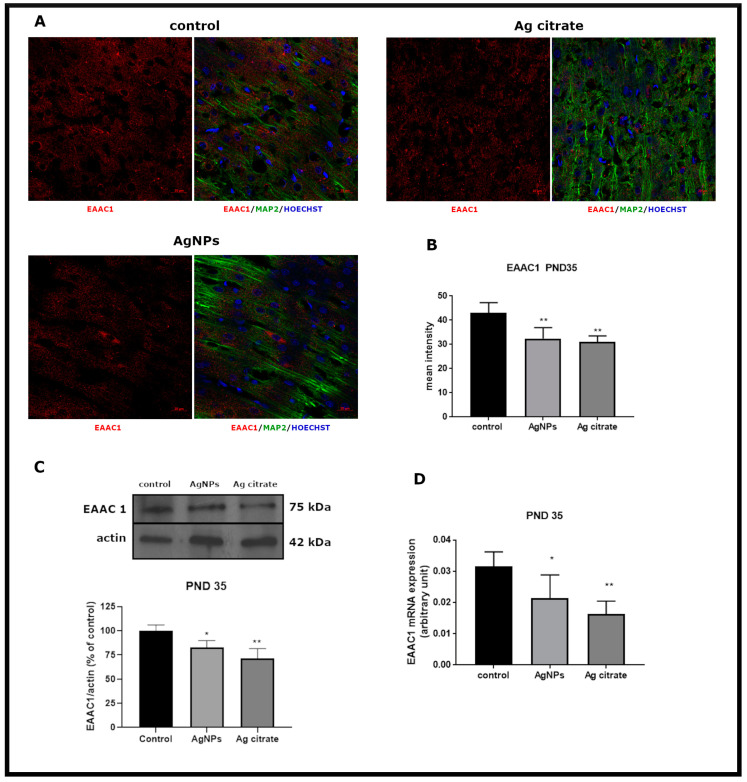
Early effects of a low dose of AgNPs/Ag^+^ on the expression of neuronal glutamate transporter EAAC1 in the brain of exposed rats (PND 35). (**A**) Representative confocal images of EAAC1 immunofluorescence in brain sections from the control and AgNP- and Ag citrate-treated rats. Double immunostaining was performed with anti-EAAC1(red) and anti-MAP-2 (green) antibodies. Nuclei were labeled with Hoechst (blue). Scale bars represent 20 μm. (**B**) The mean intensity of the fluorescence signal. Data are presented as means ± SD. ** *p* < 0.01 significantly different vs. control; n = 9–12 sections from three distinct brains in each group. (**C**) Relative expression of EAAC1 protein in the brain of the control and AgNP- and Ag citrate-treated rats. Representative immunoblot and graph illustrating the mean results of densitometric measurements of five independent immunoblots performed using four distinct animals per group. The relative density was measured against β-actin as an internal standard and expressed as a percentage of control. * *p* < 0.05, ** *p* < 0.01 significantly different vs. control. (**D**) Expression of EAAC1mRNA in the brain of the control and AgNP- and Ag citrate-treated rats. The mRNA levels were determined by quantitative real-time PCR and normalized against actin. The graph indicates the results expressed as arbitrary units from four independent experiments. * *p* < 0.05, ** *p* < 0.01 significantly different vs. control. One-way ANOVA followed by Tukey’s multiple comparison test was used for statistical analysis.

We further investigated whether there are any changes in the expression of examined GluTs in silver-exposed rats shortly after exposure (PND 35). A primary finding is that expression of neuronal transporter’s EAAC1 is significantly affected. Images obtained by confocal microscopy show decreased immunoreactivity of EAAC1 in brain sections obtained from both silver-treated groups (Figure 2A). Mean fluorescence intensity markedly declined relative to the control group (*p* < 0.01 AgNPs/Ag^+^ vs. control) (Figure 2B). Semi-quantitative W-B analysis confirmed the same trend of changes in relative concentrations of EAAC1 protein in rat brain homogenates (Figure 2C). Concomitantly with decreased protein concentrations, the level of EAAC1 mRNA was also significantly lowered in both silver-treated groups relative to the untreated control group (*p* < 0.05 and *p* < 0.01 vs. control for AgNPs and Ag citrate, respectively) (Figure 2D). Importantly, alterations in EAAC1 expression were found to persist over time.

**Figure 3:**
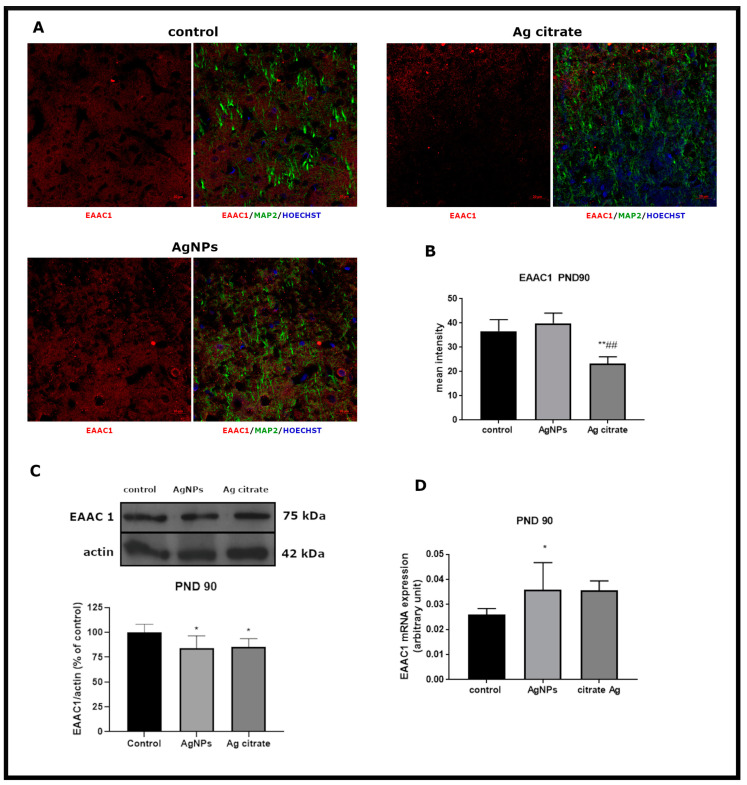
Late effects of a low dose of AgNPs/Ag^+^ on the expression of neuronal glutamate transporter EAAC1 in the brain of exposed rats (PND 90). (**A**) Representative confocal images of EAAC1 immunofluorescence in brain sections from the control and AgNP- and Ag citrate-treated rats. Double immunostaining was performed with anti-EAAC1(red) and anti-MAP-2 (green) antibodies. Nuclei were labeled with Hoechst (blue). Scale bars represent 20 μm. (**B**) The mean intensity of the fluorescence signal. Data are presented as means ± SD. ** *p* < 0.01 significantly different vs. control; ^##^ *p* < 0.01 significantly different vs. AgNP-treated rats; n = 9–12 sections from three distinct brains in each group. (**C**) Relative expression of EAAC1 protein in the brain of the control and AgNP- and Ag citrate-treated rats. Representative immunoblot and graph illustrating the mean results of densitometric measurements of five independent immunoblots performed using four distinct animals per group. The relative density was measured against β-actin as an internal standard and expressed as a percentage of control. * *p* < 0.05 significantly different vs. control. (**D**) Expression of EAAC1mRNA in the brain of the control and AgNP- and Ag citrate-treated rats. The mRNA levels were determined by quantitative real-time PCR and normalized against actin. The graph indicates the results expressed as arbitrary units from four independent experiments. * *p* < 0.05 significantly different vs. control. One-way ANOVA followed by Tukey’s multiple comparison test was used for statistical analysis.

At the PND 90 time point, the relative protein concentration was found to be consistently decreased relative to control (*p* < 0.05 AgNPs/Ag^+^ vs. control) (Figure 3C), although a compensatory increase of EAAC1 mRNA was observed in the AgNP-exposed group (*p* < 0.05 AgNPs vs. control) (Figure 3D). Immunohistochemical analysis indicated pronounced changes in the mean fluorescence intensity exclusively in Ag^+^-treated rats (*p* < 0.01 vs. control and vs. AgNPs) (Figure 3A).

**Figure 4:**
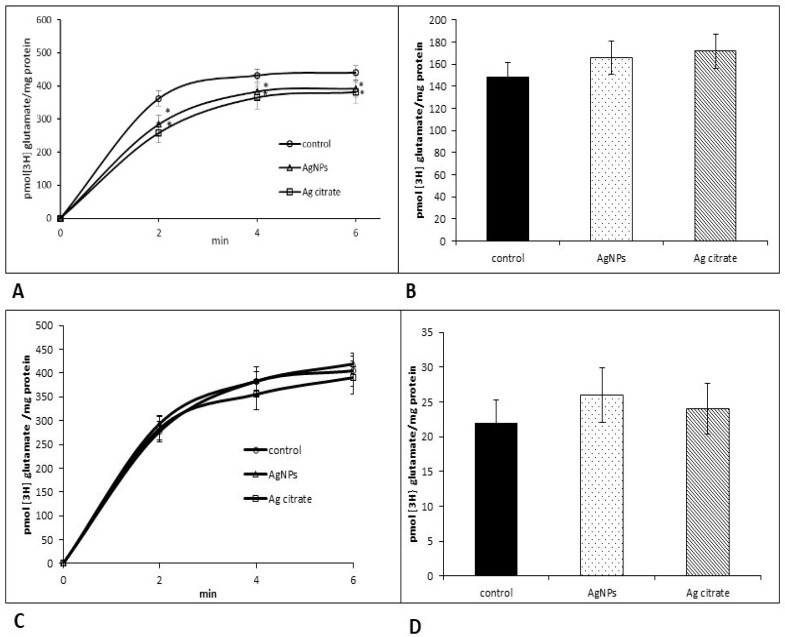
Effects of a low dose of AgNPs/Ag^+^ on Na^+^-dependent [^3^H] glutamate uptake (**A**,**C**) and release (**B**,**D**) in the synaptosomal fraction obtained from the control, AgNP- and citrate Ag-treated rats early (PND 35) (**A**,**B**) and late (PND 90) (**C**,**D**) after exposure. The results represent the mean values ± SD from four separate experiments performed in duplicate; * *p* < 0.05 significantly different vs. control. One-way ANOVA followed by Tukey’s multiple comparison test was used for statistical analysis.

Thus, it is not surprising that functional activity of transporter measured in the synaptosomal fraction was changed shortly after exposure period, in parallel with the diminished expression of EAAC1. An assay of radioactive glutamate transport revealed slightly decreased uptake of glutamate mainly in Ag^+^-exposed rats (*p* < 0.05 vs. control in all points of measurement) (Figure 4A) whereas stimulated release was not altered (Figure 4B). In AgNP-exposed animals, the amount of glutamate taken up by the synaptosomal fraction was lower relative to the control value (*p* < 0.05) only in one point of measurement, i.e., 2nd min (Figure 4A). Functional alterations were not observed later after exposure (PND 90) (Figure 4C,D).

### 2.4. Exposure to AgNPs/Ag^+^ Affects the Function but Not the Expression of Glial Glutamate Transporters GLT-1 and GLAST

**Figure 5:**
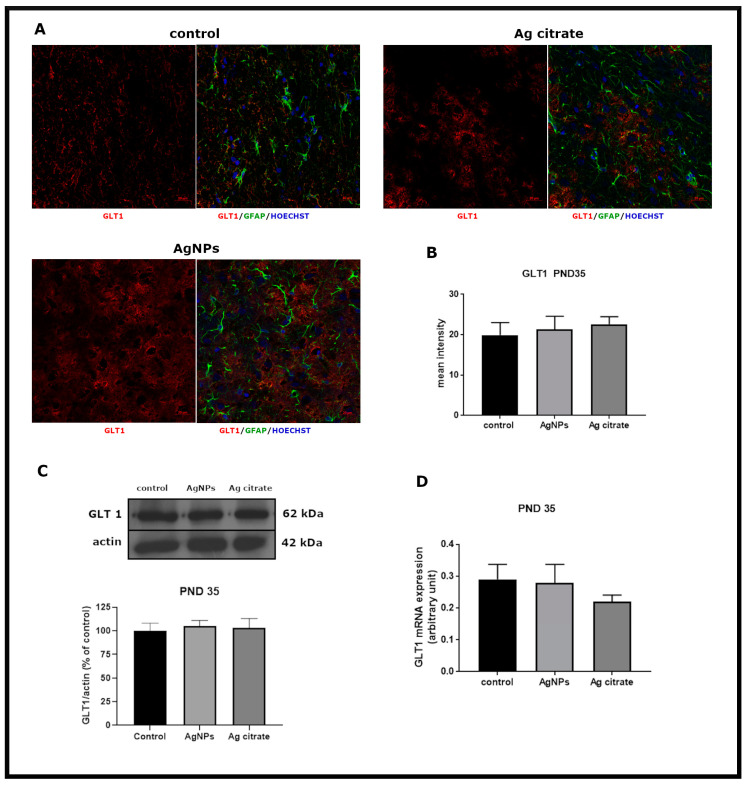
Early effects of a low dose of AgNPs/Ag^+^ on the expression of astroglial glutamate transporter GLT-1 in the brain of exposed rats (PND 35). (**A**) Representative confocal images of GLT-1 immunofluorescence in brain sections from the control and AgNP- and Ag citrate-treated rats. Double immunostaining was performed with anti-GLT-1 (red) and anti-GFAP (green) antibodies. Nuclei were labeled with Hoechst (blue). Scale bars represent 20 μm. (**B**) The mean intensity of the fluorescence signal. Data are presented as means ± SD (n = 9–12 sections from three distinct brains in each group). (**C**) Relative expression of GLT-1 protein in the brain of the control and AgNP- and Ag citrate-treated rats. Representative immunoblot and graph illustrating the mean results of densitometric measurements of five independent immunoblots performed using four distinct animals per group expressed as a percentage of control. The relative density was measured against β-actin as an internal standard. (**D**) Expression of GLAST mRNA in the brain of the control and AgNP- and Ag citrate-treated rats. The mRNA levels were determined by quantitative real-time PCR and normalized against actin. The graph indicates the results expressed as arbitrary units from four independent experiments. One-way ANOVA followed by Tukey’s multiple comparison test was used for statistical analysis.

**Figure 6:**
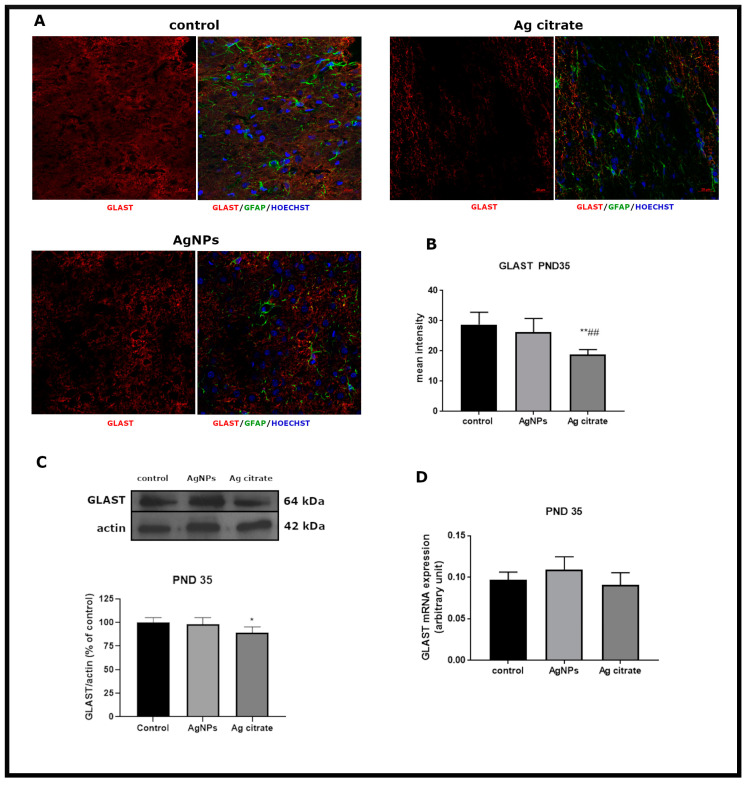
Early effects of a low dose of AgNPs/Ag^+^ on the expression of astroglial glutamate transporter GLAST in the brain of exposed rats (PND 35). (**A**) Representative confocal images of GLAST immunofluorescence in brain sections from the control and AgNP- and Ag citrate-treated rats. Double immunostaining was performed with anti-GLAST (red) and anti-GFAP (green) antibodies. Nuclei were labeled with Hoechst (blue). Scale bars represent 20 μm. (**B**) The mean intensity of the fluorescence signal. Data are presented as means ± SD. ** *p* < 0.01 significantly different vs. control, ## *p* < 0.01 significantly different vs. AgNP-treated rats (n = 9–12 sections from three distinct brains in each group). (**C**) Relative expression of GLAST protein in the brain of the control and AgNP- and Ag citrate-treated rats. Representative immunoblot and graph illustrating the mean results of densitometric measurements of five independent immunoblots performed using four distinct animals per group expressed as a percentage of control. The relative density was measured against β-actin as an internal standard. * *p* < 0.05 significantly different vs. control. (**D**) Expression of GLAST mRNA in the brain of the control and AgNP- and Ag citrate-treated rats. The mRNA levels were determined by quantitative real-time PCR and normalized against actin. The graph indicates the results expressed as arbitrary units from four independent experiments. One-way ANOVA followed by Tukey’s multiple comparison test was used for statistical analysis.

In contrast with expression of neuronal GluT, the expression of glial GluTs (GLT-1 and GLAST) was essentially unchanged immediately after the end of exposure at PND 35. Exposure to AgNPs did not influence expression of either GLT-1 or GLAST (Figure 5 and Figure 6). In the case of GLAST, a small but statistically significant decrease in protein expression and a diminished mean intensity of immunofluorescence were only observed in Ag^+^-exposed rats (Figure 6B,C).

**Figure 7:**
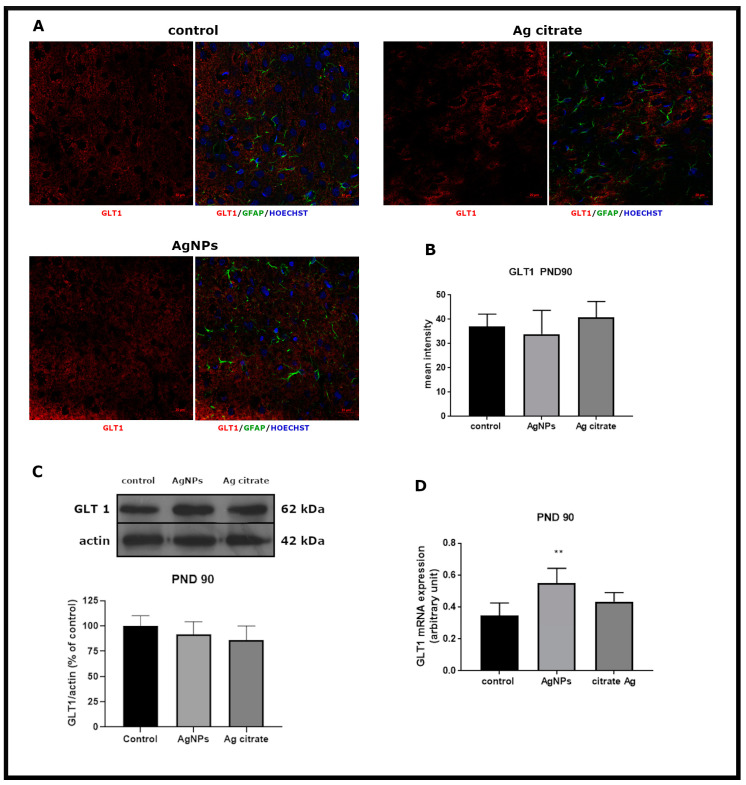
Late effects of a low dose of AgNPs/Ag^+^ on the expression of astroglial glutamate transporter GLT-1 in the brain of exposed rats (PND 90). (**A**) Representative confocal images of GLT-1 immunofluorescence in brain sections from the control and AgNP- and Ag citrate-treated rats. Double immunostaining was performed with anti-GLT-1 (red) and anti-GFAP (green) antibodies. Nuclei were labeled with Hoechst (blue). Scale bars represent 20 μm. (**B**) The mean intensity of the fluorescence signal. Data are presented as means ± SD (n = 9–12 sections from three distinct brains in each group). (**C**) Relative expression of GLT-1 protein in the brain of the control and AgNP- and Ag citrate-treated rats. Representative immunoblot and graph illustrating the mean results of densitometric measurements of five independent immunoblots performed using four distinct animals per group expressed as a percentage of control. The relative density was measured against β-actin as an internal standard. (**D**) Expression of GLAST mRNA in the brain of the control and AgNP- and Ag citrate-treated rats. The mRNA levels were determined by quantitative real-time PCR and normalized against actin. The graph indicates the results expressed as arbitrary units from four independent experiments. One-way ANOVA followed by Tukey’s multiple comparison test was used for statistical analysis. (** *p* < 0.01 significantly different vs. control).

**Figure 8:**
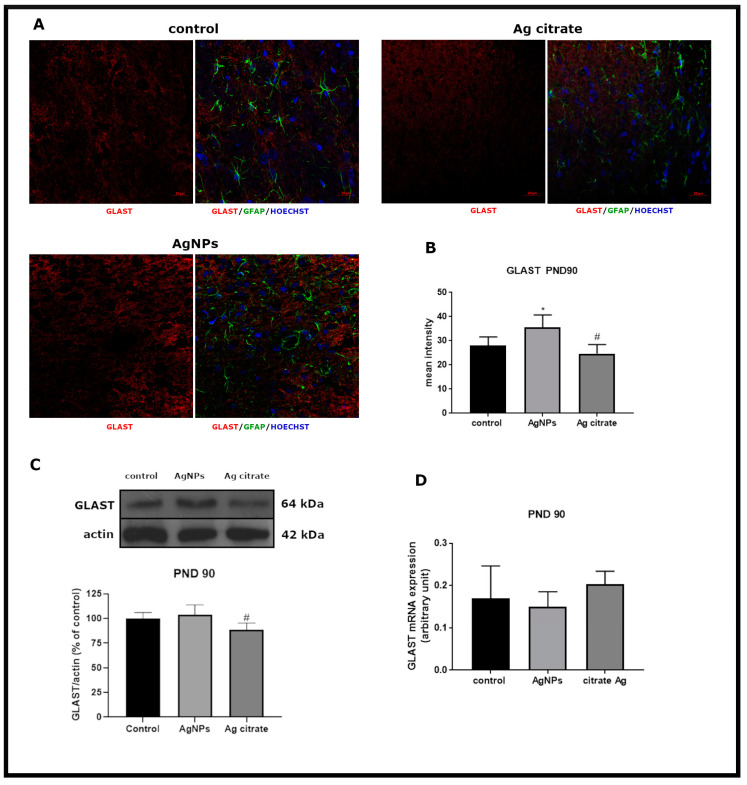
Late effects of a low dose of AgNPs/Ag^+^ on the expression of astroglial glutamate transporter GLAST in the brain of exposed rats (PND 90). (**A**) Representative confocal images of GLAST immunofluorescence in brain sections from the control and AgNP- and Ag citrate-treated rats. Double immunostaining was performed with anti-GLAST (red) and anti-GFAP (green) antibodies. Nuclei were labeled with Hoechst (blue). Scale bars represent 20 μm. (**B**) The mean intensity of the fluorescence signal. Data are presented as means ± SD. # *p* < 0.05 significantly different vs. AgNP-treated rats (n = 9–12 sections from three distinct brains in each group). * *p* < 0.05 significantly different vs. control. (**C**) Relative expression of GLAST protein in the brain of the control and AgNP- and Ag citrate-treated rats. Representative immunoblot and graph illustrating the mean results of densitometric measurements of five independent immunoblots performed using four distinct animals per group expressed as a percentage of control. The relative density was measured against β-actin as an internal standard. # *p* < 0.05 significantly different vs. AgNP-treated rats. (**D**) Expression of GLAST mRNA in the brain of the control and AgNP- and Ag citrate-treated rats. The mRNA levels were determined by quantitative real-time PCR and normalized to actin. The graphs indicate the results expressed as arbitrary units from five independent experiments. One-way ANOVA followed by Tukey’s multiple comparison test was used for statistical analysis.

However, low-level time-dependent alterations in expression of glial GluTs were present. Expression of GLT-1 mRNA was found to increase significantly in AgNP-exposed animals (*p* < 0.01 vs. control) (Figure 7D). An AgNP-dependent increase of GLAST immunoreactivity was also observed (*p* < 0.05 vs. control) (Figure 8B).

**Figure 9:**
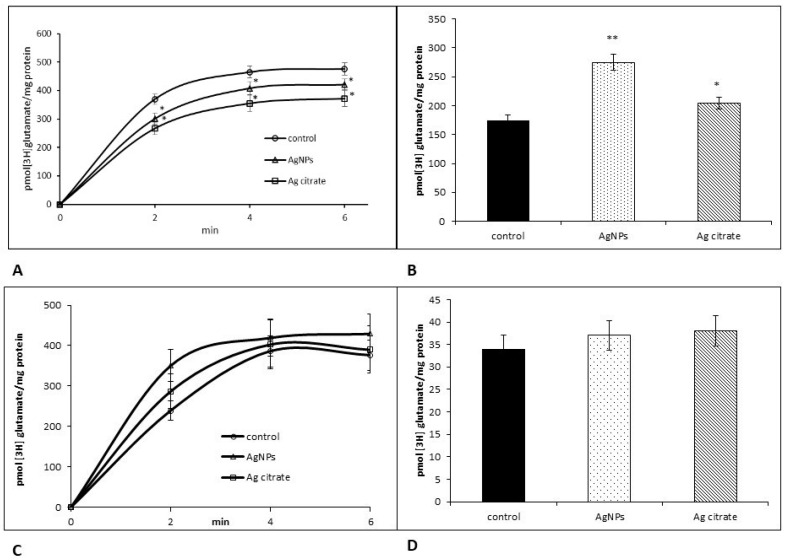
Effects of a low dose of AgNPs/Ag^+^ on Na^+^-dependent [^3^H] glutamate uptake (**A**,**C**) and release (**B**,**D**) in the glia-derived GPV fraction obtained from the control, AgNP- and citrate Ag-treated rats early (PND 35) (**A**,**B**) and late (PND 90) (**C**,**D**) after exposure. The results represent the mean values ± SD from four separate experiments performed in duplicate. * *p* < 0.05 and ** *p* < 0.01 significantly different vs. control. One-way ANOVA followed by Tukey’s multiple comparison test was used for statistical analysis.

In turn, we identified evidence of functional alterations in glial glutamate transporters shortly after exposure (Figure 9A,B) which were not persistent over a longer period (Figure 9C,D). Uptake of radioactive glutamate to the glia-derived fraction was found to be lower in both silver-treated groups (*p* < 0.05 vs. control) (Figure 9A). A significant increase of released neurotransmitter was also observed (*p* < 0.01 AgNPs/Ag^+^ vs. control) (Figure 9B).

## 3. Discussion

Neuron–glia interactions are essential for maintaining proper functioning of the synapse. For excitatory synapses, the most important function is to regulate the extracellular concentration of their neurotransmitter glutamate providing signaling which is critical for synaptic plasticity and brain development [17]. Elevated levels of glutamate and excessive stimulation of glutamatergic NMDA receptors are highly dangerous for neurons, leading finally to cell death in the process of excitotoxicity [14]. Glutamate signaling is limited by glutamate transporters (GluTs) whose proper functioning is of critical importance for preventing glutamate-induced neuronal injury. As previously reported, dysfunction and/or altered expression of these transporters underlie many pathological conditions of the CNS such as neurodegenerative diseases, epilepsy, or stroke [18,19].

### 3.1. Exposure to AgNPs/Ag^+^ during the Developmental Period Results in Stress-Like Ultrastructural Alterations in Neuronal ER and Downregulation of Neuronal Excitatory Amino Acid Carrier 1 (EAAC1)

Ultrastructural analysis revealed that neurons are targeted by silver in both the particulate (AgNPs) and the ionic forms. However, the changes are more pronounced with exposure to AgNPs. Neurons lose normal definition of subcellular structures such as nucleus, Golgi complexes (GC), and rough endoplasmic reticulum (RER). The nuclear envelope acquires an irregular surface with many invaginations as well as detachment of ribosomes from RER membranes with further degranulation. These observations provide evidence of ER stress related to neuronal injury caused by different stressors [20]. Moreover, formation of abnormally vacuolated Golgi membranes which can induce retrograde changes in RER, also provides evidence of ER stress. It is obvious that detached ribosomes cannot function normally and therefore the processes of mRNA translation and protein synthesis will be impaired. Likewise, the process of protein folding in the ER compartment and its subsequent transport to the extracellular surface may be impaired.

In light of these data, the results of expression analysis are of particular note being attributable to ultrastructural abnormalities. We show that the neuronal expression of EAAC1 is downregulated under conditions of AgNPs/Ag^+^ toxicity, presumably as a direct influence of AgNPs on neurons. EAAC1 is predominantly located intracellularly with the vast majority of the protein present inside the cell and associated with cytoplasmic vesicles, and with only a minor portion bound to the plasma membrane [21]. EAAC1 activity is modulated by post-translational mechanisms that rapidly modify its trafficking and abundance on the plasma membrane [22]. Golgi structure is responsible for packaging proteins into vesicles, which play a key role in the secretory pathway. Thus, observed ultrastructural alterations in the Golgi apparatus and ER suggest dysfunction of these cellular structures that may lead to disturbed intracellular trafficking of EAAC1 protein between the ER compartment and the plasma membrane and subsequently to the lowered membranous expression of the carrier. The ER has been shown to play a significant role in the process of transporter’s recycling. The passage through the ER and the Golgi apparatus is crucial for dynamic regulation of cell surface levels of EAAC1 [23]. Moreover, other factors triggering ER stress have also been shown to decrease both EAAC1 mRNA and protein [24]. Ultrastructural features of ER stress present in AgNP/Ag^+^-exposed animals indicate that AgNPs may act in such a mechanism. Our observation is in accordance with previous studies showing AgNP-dependent induction of ER stress-related markers (for a review, see [25]). Moreover, we also observed decreased levels of mRNA, suggesting that silver may act on multiple levels to affect the expression of the *Slc1a1* gene encoding EAAC1. The regulation of EAAC1 expression at the gene level is not characterized as thoroughly as mechanisms of transporter trafficking. Nevertheless, accumulating data indicate that the expression of the transporter on the plasma membrane is regulated at three levels: transcriptional changes in *Slc1a1* expression, changes in protein interactions, and modulation of trafficking [22]. Our TEM results suggest that direct interactions occur between both forms of silver and the nucleus of the cell. The altered shape of the nucleus with multiple invaginations of the nuclear envelope indicates disruption of the nuclear skeleton. It is known that the nuclear skeleton (or nuclear matrix) plays a structural role in the eukaryotic cell and is responsible for maintaining the shape of the nucleus and the spatial organization of chromatin. It also participates in cellular processes such as DNA replication/repair, gene expression, RNA transport, cell signaling and apoptosis [26]. Thus, AgNP-induced alterations in the nuclear matrix may affect gene expression.

Diminished EAAC1 transporter activity occurs in parallel with its lowered expression but was mainly observed under Ag^+^ exposure. EAAC1 is a subtype of glutamate transporter which is highly expressed in glutamatergic and GABAergic neurons where it fuels GABA synthesis. It does not appear to play a major role in clearance of glutamate from the extracellular space [21] but its functional role is rather related to subtle regulation of synaptic transmission [22]. It has been shown to be involved in the phenomenon of long-term potentiation (LTP) and therefore in learning and memory formation [27]. Thus, the observed downregulation of EAAC1 at both mRNA and protein levels indicates the possibility that AgNPs induce disturbances in these processes. Indeed, it has been confirmed that systemic exposure to AgNPs may result in alterations of the cerebral cognition processes in exposed mice [28]. Moreover, accumulating evidence suggests that the carrier can also transport cysteine [29,30], thereby providing an essential function in the synthesis of intracellular glutathione and subsequent protection from oxidative stress [22]. Therefore, AgNP-induced diminished expression of EAAC1 may indicate impaired regulation of cellular glutathione content and depletion of antioxidant capacity of neurons and consequently, induction of oxidative stress. The presence of markers of oxidative stress in the brain of rats exposed to a low dose of AgNPs has been previously reported [31]. The critical role of this carrier in anti-oxidative neuroprotection suggests also that such impairment of this mechanism may underlie neurodegenerative changes [32].

There is also evidence that dopaminergic neurons represent a neuronal cell population expressing EAAC1 [33] which is very sensitive to carrier dysfunction [34]. EAAC1-deficient mice present symptoms of subtle neurological damage associated with a loss of dopaminergic neurons in the substantia nigra and exhibit learning and memory dysfunction [35]. In light of these reports, it appears that EAAC1 downregulation caused by exposure to both AgNPs and Ag^+^ is substantial for developing organisms. Moreover, the results of our study partially contribute to an understanding of the mechanisms underlying behavioral dysfunctions in AgNP-exposed animals.

### 3.2. Influence of AgNPs on Glial Glutamate Transporters GLT-1 and GLAST

We obtained evidence that, unlike neuronal EAAC1, the expression levels of two astrocytic glutamate transporters, GLAST and GLT-1, are not affected by exposure to AgNPs. Both astrocytic transporters, unlike neuronal EAAC1, are specifically upregulated by the substrate (glutamate) in a dose- and time-dependent manner [36]. Astroglial GLAST expression was found to be correlated with neuronal glutamatergic activity, and glutamate has been shown to increase GLAST gene expression [37] and translocation of transporters’ protein to the plasmalemma [36,38]. Based on these data, we can assume that exposure to AgNPs/Ag^+^ does not result in enhanced glutamatergic transmission that might lead to the extracellular accumulation of glutamate and subsequent excitotoxic injury of neurons. Simultaneously, there is a lack of ultrastructural changes in astroglial cells which would suggest the existence of ER stress and hence the dysfunctionality of astrocytes. However, we observed that these transporters exhibit lower activity, as measured by the uptake of radioactive glutamate into the glia-derived fraction obtained from AgNPs/Ag^+^-exposed animals. Concomitantly with diminished uptake, the release of glutamate was found to be accelerated mainly in the rats exposed to AgNPs. This suggests that transporter systems in the glial fraction may operate partially in the reverse mode. Impairment of the transport may further lead to inefficient clearance of glutamate. The re-uptake of glutamate from the synaptic cleft into astrocytes mediated by high affinity glial transporters is an energy-dependent process which also depends on Na^+^. Na^+^-K^+^-ATPase is an enzyme that maintains an ionic gradient across the membrane, which provides a driving force for amino acid transport and is a key regulator of activity of glutamate transporters [39]. We suspect that direct or indirect interactions between AgNPs and Na^+^-K^+^-ATPase should be considered as a cause underlying dysfunctional astroglial transporters. It was previously reported that silver is the most rapid and potent inhibitor of this enzyme. The unique silver–enzyme interactions are based on an “on–off” mechanism that involves a few critical sulfhydryl groups [40]. The alternative possibility relies on indirect interactions between nanoparticles (NPs) and constituents of cellular membranes. As reported, dynamic interactions of NPs with protein and lipids tend to form a protein or lipid corona, which temporarily modulates the membrane composition, fluidity, and other characteristics [41]. Since the Na^+^-K^+^-ATPase is a membrane-bound protein, the membrane components, particularly lipids, are important determinants of enzyme function [42]. There are reports indicating that the effects of lipids on the activity of the sodium pump are related to membrane fluidity and thickness. Lipids such as phosphatidylserine or phosphatidylglycerol, which increase membrane fluidity, tend to promote Na^+^-K^+^-ATPase activity [43]. In turn, free fatty acids present in membranes or as the products of phospholipase A_2_ (PLA_2_)-dependent regulatory pathway tend to inhibit the Na^+^-K^+^-ATPase [44]. Based on the findings cited above, we hypothesized that NP-evoked alterations in membranes may underlie the observed inhibition of glial glutamate transport early after exposure. This effect is much more pronounced with AgNP exposure than with Ag^+^ exposure. Functional inhibition might then be compensated for over a longer period of time by increased expression of GLT-1 mRNA. We observed this effect in AgNP-exposed animals at the 90 PND time point (Figure 6D).

Collectively, our results demonstrate that exposure to a low dose of AgNPs/Ag^+^ in the early postnatal period leads to the persistent accumulation of silver in the brain. Regardless of its form, silver may be classified as a toxic agent affecting neurons and inducing ER stress. Selective toxic injury to neurons results in disintegration of the proper structure of the ER and its ribosomes. This may result in dysfunction of proper cellular trafficking of proteins such as glutamate transporter EAAC1. Importantly, the downregulation of this transporter at the molecular level is a long-lasting effect. Simultaneously, our results reveal that astrocytes are more resistant to AgNPs/Ag^+^ than neurons, since the expression of astroglial glutamate transporter systems GLT-1 and GLAST is unaltered and ER stress-like ultrastructural changes are not present. However, based on the previous reports, it can be stated with a high degree of probability that alterations in the activity of these transporters are caused by direct or indirect interactions with the sodium pump.

The results clearly indicate that early postnatal exposure to products containing AgNPs or Ag^+^ represents a potential risk for immature organisms, particularly when the brain is considered as a target. Our experimental assessments of the mechanisms of AgNP neurotoxicity performed in an animal model using a realistic dose and route of exposure may be useful for assessing potential health hazards of AgNPs in epidemiological studies. However, possible differences among species in metabolism and bio-interactions of AgNPs should be considered.

## 4. Materials and Methods

### 4.1. Silver Nanoparticles (AgNPs)

A homogenous suspension of AgNPs (10 ± 4 nm in diameter) in citrate buffer (conc. 0.02 mg AgNPs/mL) was purchased from Sigma-Aldrich Chemical Co. (St. Louis, MO, USA; CAS No. 730785). The manufacturer’s description indicates that each batch of AgNPs is characterized by dispersity and lack of agglomerations in a coating agent based on: refractive index, n20/D = 1.333; fluorescence, λ_em_ = 388 nm; and FWHM = 59 nm. Additional analysis of dispersion and size distribution was performed by transmission electron microscopy (TEM) using a JEM-1200EX, microscope equipped with a MORADA digital camera and iTEM 1233 software as described previously [9].

### 4.2. Animal Model of Developmental Exposure to a Low Dose of AgNPs

Experimental procedures using animals were carried out in strict accordance with the EU Directive for the Care and Use of Laboratory Animals (Directive 2010/63/EU) and in compliance with the ARRIVE guidelines and were approved by the I Local Experimental Animal Care and Use Committee in Warsaw (488/2017).

The study was conducted on two-week-old rat pups purchased from the Animal House of the Mossakowski Medical Research Centre, Polish Academy of Sciences (Warsaw, Poland). The total number of offspring, obtained from 9 eight-week-old pregnant females, was 102 (9–15 pups per litter). The exposure started from Postnatal Day 14 (PND 14). Pups, weighing 30–40 g at the beginning of the experiment, were randomly allocated into 3 groups (n = 34 pups per group): (i) an experimental group treated with AgNPs; (ii) a positive control group treated with Ag citrate as an ionic form of silver (Ag^+^); and (iii) a negative control group treated with saline. Appropriate solutions were administered to the pups once daily via a gastric tube at a dose of 0.2 mg/kg body weight (b.w.)/day for 21 consecutive days. For the first week of exposure, pups were caged with their mothers and were later separated for further exposure after starting to eat independently. Immediately after ending the exposure, at Postnatal Day 35 (PND 35 group), half of the animals from each of the three groups was selected for biochemical, molecular and microscopic analyses. The remaining animals were caged until Postnatal Day 90 (PND 90 group).

### 4.3. Determination of Silver Concentration with Inductively-Coupled Plasma Mass Spectrometry (ICP-MS)

Rats were sacrificed by decapitation at PND 35 and PND 90, respectively, and samples of blood and tissues were taken from control and exposed groups. Blood was collected and centrifuged to obtain serum (1000× *g*, 4 °C, 10 min) which was further diluted with 1% Triton. Whole brains were weighed, lyophilized, and digested with concentrated nitric acid. Silver concentrations were measured using ICP-MS (Elan 6100 DRC Sciex Perkin Elmer, Markham, ON, Canada). Three concentrations of internal standard (ICP Multielement Standard, TraceCERT(R), Sigma Aldrich) were used to calculate silver concentrations in the samples.

### 4.4. Analysis of Gene Expression by qPCR

Early or late after exposure, at PND 35 or PND 90, respectively, brains were quickly removed under sterile conditions, placed in liquid nitrogen and stored at −80 °C for further analysis.

Total RNA was extracted from the brain cortex of the rats in all experimental groups. Isolation was performed using TRI Reagent (Sigma-Aldrich, St. Louis, MO, USA) according to the method of Chomczyński [45]. Total RNA concentration was determined using DS-11Fx nano-spectrophotometer/fluorometer (De Novix, Wilmington, DE, USA). Two micrograms of total RNA were reverse-transcribed using random primers and AMV reverse transcriptase (Life Technologies, Forest City, CA, USA). The RT-PCR conditions included reverse transcription 42 °C for 45 min followed by denaturation at 94 °C for 30 s. TaqMan assays were employed for quantitative real time PCR analysis. The specific primers for rat excitatory amino acid transporters: GLAST Rn 00570130_m1 (gen Sl1a3); GLT-1 Rn 00691548_m1 (gen Slc1a2) and EAAC1 Rn 00564705_m1 (gen Slc1a1) were used. Actin (*Actb)* (Rn 00667869_m1) was used as a reference gene. The probes were obtained from Life Technologies (Forest City, CA, USA). To normalize the mRNA expression, levels of EAATs and actin were determined using the TaqMan assay reagents (Life Technologies, Forest City, CA, USA). Quantitative real time PCR (qPCR) analysis was conducted on a Roche LightCycler^®^ 96 system, using 5 μL of RT product and TaqMan PCR Master Mix, primers, and TaqMan probe in a total volume of 20 μL. Cycle conditions for the qPCR were as follows: initial denaturation at 95 °C for 10 min and 50 cycles of 95 °C for 15 s and 60 °C for 1 min. Each sample was analyzed in triplicate. The relative expression levels of EAATs were calculated on basis of the _ΔΔ_Ct method.

### 4.5. Western Blot Analysis

Brain tissue was homogenized in 50 mM phosphate buffer pH 7.4, containing 10 mM EGTA, 10 mM EDTA, 0.1 mM PMSF, and 100 mM NaCl in the presence of a protease inhibitor cocktail (1 µg/mL leupeptin, 0.1 µg/mL pepstatin, and 1 µg/mL aprotinin) and used in an immunoblotting assay. Samples of 30–50 μg of protein/lane were mixed with loading buffer, subjected to 10% SDS-PAGE under reducing conditions, and then transferred onto a nitrocellulose membrane (Hybond-ECL, Amersham, UK). After appropriate blocking steps (PBS/5% milk for 90 min), blots were incubated overnight at 4 °C with primary antibodies: polyclonal anti-GLAST, 1:1000 (Abcam, Cat. No. ab 416), polyclonal anti-GLT1, 1 µg/mL (Abcam, Cat. No. ab 41621), and monoclonal anti-EAAC1, 1:1000 (Abcam, Cat. No. ab 124802). Then, the membranes were incubated with the secondary antibody conjugated with HRP (Sigma, Cat. No. A-9169, 1:5000). Monoclonal anti-actin antibody (Abcam, Cat. No. ab 3280, 0.5 µg/mL) was used as internal standard. Bands were visualized using the ECL kit and Hyperfilm ECL (Amersham). Autoradiography films were converted into 8-bit grayscale digital files using ImageScanner III (GE Healthcare, LabScan 6.0 software). Densitometric analysis was performer with ImageQuant TL software version 2.4.

### 4.6. Preparation of Brain Fractions

A fraction containing nerve endings (synaptosomes) and a glia-derived fraction (glial plasmalemmal vesicles; GPV) were prepared from freshly isolated brain tissue. The brain tissue was homogenized in 30 mL of isolation medium (0.32 M sucrose and 1 mM EDTA) and centrifuged at 1000× *g* for 10 min. Supernatant was then applied in further procedures.

To obtain a synaptosomal fraction using the method of Booth and Clark [46], the supernatant was centrifuged in a discontinuous Ficoll gradient (7%, 12%) at 99,000× *g*. The synaptosomal pellet was washed once in Krebs-Ringer buffer at pH 7.4 (140 mM NaCl, 5 mM KCl, 10 mM Tris-HCl, 1.4 mM MgSO_4_, and 1 mM Na_2_HPO_4_) and suspended in the same buffer to obtain a protein concentration of approximately 5 mg/mL.

To obtain GPV fractions by the method of Daniels and Vickroy [47], the supernatant was diluted using SEDH medium (0.32 M sucrose, 1 mM EDTA, 0.25 mM dithiothreitol, and 20 mM HEPES, pH 7.4) and centrifuged at 5000× *g* for 15 min. After several additional fractionations, the material was centrifuged in a three-step discontinuous Percoll gradient (20%:10%:6%) for 10 min at 33,500× *g*. The layer between 0% and 6% Percoll was collected to obtain the GPV. Both fractions were used for [^3^H] glutamate transport (uptake and release) assays.

### 4.7. [^3^H] Glutamate Transport Assay

Assays of Na^+^-dependent [^3^H] glutamate uptake and KCl-dependent release of accumulated amino acid were performed using freshly isolated synaptosomal or GPV fractions. The protein concentration in both fractions was determined by the method of Lowry et al. [48]. Radioactive glutamate accumulation in isolated fractions was performed according to the filtration method described by Divac [49]. Radioactivity trapped on the filters was then measured in a liquid scintillation counter (Wallack 1409). At a maximum of the uptake curve (6 min), 50 mM KCl was added as an agent stimulating the release of previously accumulated neurotransmitter. Liberated radioactivity was assayed after 6 min. To prevent the metabolism of glutamate in the mixture, aminooxyacetic acid (AOAA) was added to inhibit aspartate aminotransferase (AAT) in order to prevent enzymatic conversion of glutamate to α-ketoglutarate [50].

### 4.8. Immunohistochemical Procedure and Microscopic Analysis

Rats were anesthetized with a lethal dose of Vetbutal and perfused through the heart with phosphate-buffered saline (PBS) and 4% paraformaldehyde in PBS. Brains were cryoprotected by immersion of tissue samples in increasing concentrations of sucrose solutions (10%, overnight; 20%, 2 days; and 30%, 5 days). Next, brains were frozen to cut into sections (20-μm thick) on a cryostat (Leica CM1860 UV, Leica Biosystems, Nussloch, Germany) and placed on slides (ThermoScientific^TM^ Superfrost Plus, Sigma-Aldrich, Poznań, Polska).

Coronal brain sections were co-stained using either primary rabbit polyclonal antibody against GLAST (1:100; Cat. No. ab 416, Abcam) together with mouse primary antibody against GFAP (1:100; Sigma Cat. No. G 3893), rabbit polyclonal anti-GLT1 (1:500; Abcam Cat. No. ab 41621) together with the mouse primary antibody against GFAP (1:100; Sigma Cat. No. G 3893), or rabbit monoclonal anti-EAAC1 (1:500; Abcam Cat. No. ab 124802) together with the mouse primary antibody against MAP2 (1:750; Sigma Cat. No. M 4403). Then, the secondary goat anti-mouse antibodies conjugated with Alexa Fluor 488 (1:500; Invitrogen Cat. No. A 21121) and the secondary goat anti-rabbit antibodies conjugated with Alexa Fluor 546 (1:500; Molecular Probes Invitrogen Cat. No. A11010) were added and the slides were exposed for 60 min in the dark. In addition, cell nuclei were stained with Hoechst 33258 (1 μg/mL; Sigma-Aldrich Cat. No. B2261). After washing with PBS, the slides were mounted with Fluorescent Mounting Medium (Dako Denmark A/S, Glostrup, Denmark). Control of immunostaining specificity was established by the omission of the primary antibodies in the incubation mixture. After staining, the slides were examined using the LSM 780/ELYRA PS.1 super-resolution confocal system (Carl Zeiss, Jena, Germany). Mean fluorescence intensity on micrographs was measured using ZEN 2.6 software (Carl Zeiss, Jena, Germany).

Microscopic analysis was performed in Laboratory of Advanced Microscopy Techniques, Mossakowski Medical Research Centre, Polish Academy of Sciences, Warsaw, Poland.

### 4.9. Ultrastructural Analysis by TEM

Rats were anesthetized with a lethal dose of Vetbutal and perfused through the ascending aorta with 0.9% NaCl in 0.01 M sodium-potassium phosphate buffer (pH 7.4) and then with 2% paraformaldehyde and 2.5% glutaraldehyde in 0.1 M cacodylate buffer (pH 7.4). Samples of brains were taken, fixed in the above fixative solution, and then post-fixed in 1% OsO_4_ solution. The material was dehydrated in the ethanol gradient, embedded in epoxy resin (Epon 812), and cut into ultra-thin sections. Next, sections were stained with 9% uranyl acetate and lead nitrate and analyzed by TEM (JEM-1200 EX, Jeol, Japan). Images were taken with a MORADA camera and iTEM 1233 software (EMSIS GmbH, Germany). 

### 4.10. Statistical Analysis

The results of the analyses are presented as means ± SD from the number of experiments indicated under the each of figures. One-way analysis of variance (ANOVA) followed by Tukey’s or Bonferroni’s (Ag concentration) multiple comparisons tests was applied to assess differences between groups at a *p* level < 0.05. GraphPad Prism Software, version 6.0 (San Diego, CA, USA) was used as a statistical tool.

## Figures and Tables

**Table 1:** Temporal profile of silver concentration in serum and brain of rats exposed to a low dose of particulate or ionic silver (AgNPs/Ag^+^) for three weeks as measured by ICP-MS.

Group	Silver Concentration			
	Serum (µg/L)		Brain (mg/kg w.w.)	
	Short-Term (PND 35)	Long-Term (PND 90)	Short-Term (PND 35)	Long-Term (PND 90)
Control	1.10 ± 0.79	<DL^1^	<DL^2^	<DL^2^
AgNPs	22.57 ± 5.57 ****	0.42 ± 0.06 ^####^	0.15 ± 0.01 ***	0.20 ± 0.03 **
Ag citrate	23.23 ± 4.83 ****	0.40 ± 0.14 ^####^	0.23 ± 0.03 ***^,+++^	0.53 ± 0.11 ****^,+++^

Values are means ± SD from 3–4 independent samples; ** *p* < 0.01, *** *p* < 0.001, **** *p* < 0.0001 vs. control in respective columns; ^####^ *p* < 0.0001 vs. the respective value in PND 35; ^+++^ *p* < 0.001 vs. AgNPs in respective columns (one-way ANOVA with post hoc Bonferroni’s test); DL^1^, detection limit for serum = 0.190 µg/L; DL^2^, detection limit for solid tissue 0.011 mg/kg wet weight (w.w.).
